# Study on Mechanisms Underlying Changes in Agricultural Carbon Emissions: A Case in Jilin Province, China, 1998–2018

**DOI:** 10.3390/ijerph18030919

**Published:** 2021-01-21

**Authors:** Hongpeng Guo, Boqun Fan, Chulin Pan

**Affiliations:** College of Biological and Agricultural Engineering, Jilin University, 5988 Renmin Street, Changchun 130022, China; ghp@jlu.edu.cn (H.G.); fanbq19@mails.jlu.edu.cn (B.F.)

**Keywords:** China Jilin Province, agricultural carbon emissions, carbon emission influential factors, LMDI decomposition model, strategy for carbon emission reduction

## Abstract

Reducing agricultural carbon emissions (ACE) is a key point to achieve green and sustainable development in agriculture. Based on the ACE statistics of Jilin Province in China from 1998 to 2018, this article considers the sources of ACE in depth, and fourteen different carbon sources are selected to calculate ACE. Besides, the paper explores the variation characteristics of ACE in Jilin Province, their structure, and the relationship between the intensity and density of the dynamic changes in ACE in the province in terms of time. Finally, this paper uses the Kaya identity and logarithmic mean Divisia index (LMDI) to analyze the influential factors in ACE. The results show the following: (1) During 1998–2018, the amount of ACE in Jilin Province increased, with an average annual growth rate of 1.13%. However, the chain growth rate has been negative in recent years, which reflects that carbon emission reduction has been achieved to a certain extent. (2) The characteristics of ACE in Jilin Province during the years is that of the low-intensity, high density category. Furthermore, agricultural resource input is the main source of the planting industry’s carbon emissions. From the perspective of animal husbandry, the proportion of CH_4_ decreased, while the proportion of N_2_O is relatively stable. (3) Based on the LMDI decomposition model, production efficiency, industrial structure, and labor are the three main factors that reduce ACE in Jilin Province. The economic level is the main factor of ACE, and it will be the most important factor leading to an increase in ACE in the short term. On the basis of comprehensive analysis, this article puts forward reasonable suggestions in terms of policy improvement, production mode and industrial structure adjustment, technological innovation, and talent introduction.

## 1. Introduction

Since the beginning of the 21st century, the carbon emissions caused by human activities have increased year by year, which has had many negative effects on the productivity and life of human beings. At present, carbon emissions are a key reason for climate change [1]. China is not only the largest developing country in the world but also the largest country that emits carbon dioxide [2]. To reduce the negative impact of global warming and the deterioration of the ecological environment, China, as one of the important member states of the United Nations, at the 2009 Copenhagen Conference, made a commitment to reduce carbon dioxide emissions per unit of Gross Domestic Product (GDP) by 40%–45% from the 2005 level by 2020 [3]. Afterward, at the Climate Change Conference in Paris in 2016, the Chinese government promised to make carbon dioxide emissions peak by the end of 2030 and to strive to achieve the goal as early as possible. According to the report of the United Nations Intergovernmental Panel on Climate change (IPCC), agricultural production has become the second-largest source of greenhouse gas emissions in the world, accounting for 14% of the world’s greenhouse gas emissions. In addition, agricultural emissions account for 50% of CH_4_, 70% of N_2_O, and 20% of CO_2_ [4]. Therefore, reducing agricultural carbon emissions, as an important source of greenhouse gas emissions, is essential.

Jilin Province, located in northeast China [5], is a major agricultural province in China and is located in the world-famous black soil belt. The total amount of cultivated land ranks fifth in the country. According to the Statistical Bulletin on National Economic and Social Development in 2019, the total grain output of Jilin Province reached 38.78 million tons in 2019 and remained at more than 35 million tons for seven consecutive years, ranking first in the country in net increase. Therefore, it is of great significance to China to develop low-carbon agriculture and sustainable agriculture based on relevant studies of existing problems in agricultural carbon emissions in Jilin Province.

The term agricultural carbon emissions is what we refer to as greenhouse gas emission caused directly or indirectly by chemical fertilizers, pesticides, fossil fuels, and waste disposal in the process of agricultural production [6]. In previous studies, most scholars have estimated agricultural greenhouse gas emissions generally based on Volume 4 of the 2006 IPCC Guidelines on National Greenhouse Gas Inventories [7,8,9,10,11]. However, different scholars have different opinions and use different methods of calculating. For example, Bennetzen et al. (2016) [12] applied the Kaya–Porter identity (KPI) to calculate greenhouse gas emissions from farming and animal husbandry in global agricultural production and predicted possible changes in the future. Peter et al. (2017) [13] used calculators to assess greenhouse gas emissions from crop cultivation, while Linderholm (2020) [14] adopted carbon capture Life Cycle Assessment (CC-LCA), an approach that calculates the carbon footprint of agricultural production and the net greenhouse gas emissions of agricultural production, thereby making the scope of consideration more comprehensive.

In recent years, scholars have analyzed greenhouse gas emissions from different perspectives, believing that such emissions are influenced by different carbon emission sources and various factors. On the one hand, many scholars have pointed out that the use of chemicals in agricultural production and life, such as chemical fertilizers, pesticides, and agricultural films, produces certain carbon emissions [15]. In addition, energy consumption, such as irrigation electricity and fossil fuel combustion, also lead to an increase in carbon emissions [16]. Furthermore, land-use change also produces a lot of carbon emissions [17,18]. On the other hand, rice cultivation; manure of cattle, sheep, goats, pigs, chickens, ducks, and other livestock and poultry; and straw burning are also the main factors that cause carbon emissions in the process of crop production [9,19,20,21,22,23]. Due to the diversity of carbon sources to a certain extent, different scholars have adopted a variety of empirical methods of studying the influential factors. The methods commonly applied are the Kaya identity, the logarithmic mean Divisia index (LMDI) decomposition model, the environmental Kuznets curve (EKC), the co-integration autoregressive distributed lag (ARDL) model, the nonlinear autoregressive distributed lag (NARDL) co-integration technique, the STIRPAT model, the denitrification decomposition (DNDC) online model, and so on [24,25,26,27,28,29]. Due to specific characteristics of the LMDI model, such as reversible factorization, complete decomposition, and no residuals in decomposition results, the LMDI model is the selected model for this paper.

Some of the previous studies were are mainly on how to reduce carbon emissions, a hot topic in the research on the field recently. First, carbon emissions can be reduced by government policies, such as modifying greenhouse gas emission caps, optimizing management practices, increasing carbon taxes, and increasing regulatory efforts [30,31,32]. Second, carbon emissions can be mitigated by using green fertilizers, avoiding crop burning, adjusting the soil’s physical and chemical properties, and minimizing agricultural resource inputs so as to achieve a circular and sustainable ecological environment [33,34,35]. Finally, low-carbon agriculture can be further achieved by adopting conservation tillage, enhancing the use of agricultural input resources, facilitating the optimization and upgrading of the industrial structure, and introducing new technological means such as biochar [36,37,38,39,40].

By reviewing the existing literature, many scholars have analyzed the issues related to agricultural carbon emissions from different perspectives. At present, the research on agricultural carbon emissions mainly focuses on the following three aspects: the first is the measurement methods of agricultural carbon emissions, the second is the influential factors in agricultural carbon emissions, and the third is the countermeasures of agricultural carbon emission reduction. There are many studies on carbon emissions at present, but they focus on only one aspect of planting or animal husbandry, and there is a lack of overall research on agricultural carbon emissions in Jilin Province, a major agricultural province in China. Therefore, based on what has been achieved in previous relevant studies so far, this paper takes Jilin Province of China as an example, studies the current situation of agricultural carbon emissions in Jilin Province, and calculates the total carbon emissions from planting and animal husbandry in the 21 years from 1998 to 2018. Additionally, through dynamic analysis of the change characteristics of agricultural carbon emissions, structural changes, agricultural carbon emission intensity, and density, finding out the changes in agricultural carbon emissions. Finally, through the construction of the logarithmic mean Divisia index (LMDI) factor decomposition model, the main driving factors in agricultural carbon emissions in Jilin Province are identified, and relevant suggestions can be put forward on the development of low-carbon agriculture in Jilin Province. Different from all the other studies, the contribution of this study can be seen as follows: First, the research is done by taking both planting and animal husbandry as a new joint basis. Second, this paper is more comprehensive in selecting carbon sources, with fourteen carbon emission sources being included in the collection, which improves the accuracy of agricultural carbon emission measurement. By using the expanded Kaya identity, the influential factors in agricultural carbon emissions are classified as production efficiency factors, economic-level factors, industrial structure factors, and labor factors. Furthermore, the LMDI is applied to investigate the impact of various factors on agricultural carbon emissions. This can be used as a reference for other provinces in China and even the whole world, providing data support and an empirical basis for the formulation of policy recommendations on agricultural carbon emissions in other countries and regions.

## 2. Materials and Methods

### 2.1. Data

The samples in the case study of agricultural carbon emissions in the paper are selected from a database between 1998 and 2018, which is 21 years in total. The data of the various indicators analyzed are derived from three different yearbooks: the China Statistical Yearbook, the Jilin Province Statistical Yearbook, and the China Rural Statistical Yearbook. In them, the amounts of chemical fertilizers, pesticides, and agricultural plastic films are what have been actually used in those years. The size of the land plowed is subject to the area sown with crops in the current year. Agricultural irrigation is based on the effective irrigated area in the current year. The sown area of rice is taken as the actual sown area of that year.

### 2.2. Estimating Agricultural Carbon Emissions

Agricultural carbon emissions are a significant criterion for measuring the level of sustainable agricultural development and low-carbon agriculture. The measurement and calculation of agricultural carbon emissions are mainly based on the carbon emission coefficient method. For the planting industry, carbon emission sources were mainly studied from seven aspects: fertilizers (convert into purity), agricultural diesel fuel, agricultural plastic films, pesticides, land plowing (the real sown area of crops), irrigation, and paddy rice planting.

For animal husbandry, carbon emissions caused by animal intestinal fermentation and manure emissions are also one of the important sources of agricultural carbon emissions (ACE). Carbon emission sources in animal husbandry include cattle, horses, donkeys, mules, goats, sheep, and pigs. In the paper, three different greenhouses gases, CO_2_, CH_4_, and N_2_O, were converted to CO_2_eq using the global warming potential (GWP) for the convenience of calculation and analysis. Specifically, CO_2_eq is a unit of measure of different greenhouse gas emissions that can be used to standardize the greenhouse effects of different greenhouse gases, including carbon-based greenhouse gases such as carbon dioxide and methane, and non-carbon greenhouse gases such as nitrous oxide. Therefore, the database for the study is taken from the assessment report of the United Nations Intergovernmental Joint Committee on Climate Change (IPCC), and research on the measurement and calculation of agricultural carbon emissions is uniformly conducted using CO_2_eq. Therefore, GWP (CO_2_) = 1, GWP (CH_4_) = 25, and GWP (N_2_O) = 298, that is, 1 ton of methane equals 25 tons of carbon dioxide, and 1 ton of nitrous oxide equals 298 tons of carbon dioxide.

#### 2.2.1. Carbon Emission Calculation for the Planting Industry

The carbon emission calculation for the planting industry can be performed using the following Equation (1) based on what Tian and Zhang proposed (2013) [41]:

(1) E=∑Ci=∑Ti×δi,

where E represents the total agricultural carbon emission, i represents the type of carbon source, Ci is the carbon emission of the $i_{\mathrm{th}}$ type of carbon source, Ti is the used amount of the $i_{\mathrm{th}}$ type of carbon source, and δi is the coefficient of the $i_{\mathrm{th}}$ type of carbon source.

The carbon emission coefficient of each carbon source is shown in Table 1. The coefficients are derived from classic studies in the field of natural science (Wu et al., 2002, IPCC 2007, Tian 2013). These studies have been widely accepted and applied, and their accuracy has been recognized as well.

#### 2.2.2. Carbon Emission Calculation for the Animal Industry

According to the IPCC’s Fifth Assessment Report (2014) [53], the measurement and calculation of agricultural carbon emissions are represented with CO_2_eq uniformly, so the calculation of total carbon dioxide emissions in animal husbandry in Jilin Province is as shown in Equation (2):

(2) $$E_{CO_{2}}=25E_{CH_{4}}+298E_{N_{2}O}$$

Methane or N_2_O emissions per animal species in Jilin Province are measured as the product of the year-end number of animals (AP; head/year) and their emission factor (EF; kg/unit/year), as shown in Equation (3):

(3) $$E_{i}=EF\times AP_{i}\times 10^{-7},$$

where $E_{i}$ represents the amount of methane or nitrous oxide emissions produced by animal husbandry. If i = CH_4_, then $E_{i}$ represents the methane emissions of different animals. Besides, if i = N_2_O, then $E_{i}$ represents the nitrous oxide emissions of different animals (unit: 10,000 tons/year).

The carbon source coefficients of various animals in the livestock industry are shown in Table 2.

### 2.3. The Intensity and Density of Agricultural Carbon Emissions

The total amount of agricultural carbon emissions in Jilin Province can be calculated based on what is shown above, and the intensity and density of agricultural carbon emissions can be calculated in Jilin Province using Equation (4). The carbon intensity refers to the carbon emissions generated by the growth of the GDP per unit, which is mainly used to evaluate the relevance between economic development and carbon emissions in a region.

(4) $$Cg=\frac{\sum E_{i}\times \beta_{i}}{G},$$

where $C_{g}$ represents the intensity of agricultural carbon emissions, in unit of tons/ten thousand yuan; $E_{i}$ represents the input of seven kinds of supplies, such as fertilizers and agricultural plastic films; $\beta_{i}$ represents the carbon emission coefficient of the i supplies; and G represents the total output value of agriculture and animal husbandry.

What we call the agricultural carbon emission density actually refers to the amount of carbon emissions per unit planting area. The agricultural carbon emission density is calculated by Equation (5).

(5) $$A=\frac{E}{B}=\frac{\sum e_{i}}{B}=\frac{\sum T_{i}\times \delta_{i}}{B}$$

### 2.4. Influential Factors in Agricultural Carbon Emissions

Up to now, for research on the influential factors in carbon emissions, decomposition analysis methods have been mostly applied. Decomposition analysis consists of the following two methods: structural decomposition analysis (SDA) and index decomposition analysis (IDA). Compared to the SDA, the IDA’s advantages in practical applications are that it does not require input–output table data but only needs to analyze and add time series data. Therefore, it is more feasible for data collection in the early stage, which is consistent with the research in this paper. What the IDA emphasizes on is to break down target variables into several different combinations of influencer factors for research. The Kaya identity and the LMDI model applied in IDA analysis in this paper will be introduced next.

#### 2.4.1. Kaya Identity

The Kaya identity was originally proposed by the Japanese scholar Yoichi Kaya at the IPCC meeting in 1990. It is mainly used to analyze the degree of correlation between carbon dioxide emissions caused by human activities and the population size, economy, and policy [54]. At present, the Kaya identity is widely used by researchers in the study of driving factors causing changes in agricultural carbon emissions in different countries and regions. This paper is based on the basic theory of Kaya identities, combined with the reality in Jilin Province, to modify the Kaya identities, as shown in Equation (6):

(6) $$\begin{array}{cc} C= & \frac{C}{AGDP}\times \frac{AGDP}{PGDP}\times \frac{PGDP}{AL}\times AL\\ & \;=EI\times SI\times CI\times AI, \end{array}$$

where C represents agricultural carbon emissions, referring to the total agricultural carbon emissions in Jilin Province; AGDP represents the total output value of agriculture and animal husbandry; PGDP represents the total output value of agriculture, forestry, animal husbandry, and fishery; AL represents the total agricultural labor force; EI represents agricultural production efficiency indicators; SI represents agricultural structural factors; CI represents agricultural economic development-level factors; and AI represents agricultural labor factors.

#### 2.4.2. LMDI Decomposition Model

As for the LMDI model, its full name is the logarithmic mean Divisia index decomposition method, a kind of IDA index decomposition method. Compared to other index decomposition methods, this method has the advantages of complete decomposition, reversible factor decomposition, no residuals in decomposition results, and zero values allowed in the data. Therefore, it is reasonable and feasible to choose the LMDI model. This paper takes the 1998 data as the base period, based on the research content; sets the total agricultural carbon emissions in the base period as $C^{0}$ and the total agricultural carbon emissions in period T as $C^{T}$; and takes the subscript TOT to represent the overall change. The formula is as follows:

(7) $$\Delta C_{TOT}=C^{T}-C^{0}$$

By means of the addition decomposition method, the decomposition of the total effect of changes in agricultural carbon emissions, as shown in Formula (8)

(8) $$\Delta C_{TOT}=\Delta EI+\Delta SI+\Delta CI+\Delta AI$$

Therefore, the expressions of contribution values of each decomposition factor are

(9) $$\begin{array}{c} Y=\sum \frac{C^{T}-C^{0}}{\ln C_{i}^{T}-\ln C_{i}^{T}}\\ \Delta EI=Y\times \ln\frac{EI^{T}}{EI^{0}} \end{array}$$

(10) $$\Delta SI=Y\times \ln\frac{SI^{T}}{SI^{0}}$$

(11) $$\Delta CI=Y\times \ln\frac{CI^{T}}{CI^{0}}$$

(12) $$\Delta AI=Y\times \ln\frac{AI^{T}}{AI^{0}}$$

## 3. Results and Discussion

### 3.1. Estimation Results and Analysis of Emissions

#### 3.1.1. Analysis of the Dynamic Changes in the Agricultural Carbon Emission Structure

According to Equation (1), the total carbon emissions from the planting industry in Jilin Province were worked out in this study, as shown in Table 3. Currently, the carbon emissions from the planting industry in Jilin Province are gradually increasing year by year, showing a slowly rising trend, with the emissions increasing from 1.684 million tons in 1998 to 3.4662 million tons in 2018, an increase of 1.7822 million tons, with an average annual growth rate of 3.80%. Judging from the average level over the past 20 years in Jilin Province, the carbon emissions caused by the use of chemical fertilizers are the highest, accounting for 57.61% of the total carbon emissions from the planting industry, and the carbon emissions caused by rice cultivation have increased. The percentages of agricultural diesel, agricultural plastic films, pesticides, land plowing, and effective irrigation were 11.29%, 9.7%, 7.21%, 0.06%, and 0.02%, respectively. The main reason is that since agriculture is the main industry in Jilin Province and rice is regarded as one of the main food crops, the sown area of rice and its corresponding output increase annually with the repaid development of the economy, which causes an increase in carbon emissions.

Based on Equations (2) and (3), the total carbon emissions from animal husbandry in Jilin Province could be worked out, as shown in Table 4. Animal husbandry is an important source of agricultural carbon emissions in Jilin Province, China. Emissions decreased by 1.637 million tons from 10.2611 million tons in 1998 to 8.6141 million tons in 2018. Especially in recent years, with the widespread use of agricultural machinery and equipment, the number of animals has decreased, which has correspondingly reduced the carbon emissions produced by intestinal fermentation and feces of livestock to a certain extent. At present, methane and nitrous oxide are the two major sources of non-carbon greenhouses in Jilin Province. Among agricultural greenhouse gases in animal husbandry in Jilin Province, CH_4_ has the largest proportion, but its proportion is shrinking day by day. The proportion of N_2_O is relatively stable. Methane (CH_4_) and nitrous oxide (N_2_O) account for 65% and 35% of carbon emissions from animal husbandry, respectively. Among them, cattle and sheep account for the largest proportion of carbon emissions caused by intestinal fermentation and fecal management. As for donkeys and mules, since they constitute a smaller proportion of carbon emissions, they are put into one category for study. The average emissions of methane and nitrous oxide from cattle are the highest. However, with the promulgation of national policies and regulations and the consistent adjustment of the industrial structure of animal husbandry, carbon emissions from animal husbandry are showing a decreasing trend.

#### 3.1.2. Analysis of Temporal Variations in Agricultural Carbon Emissions in Jilin Province

In this section, the total agricultural carbon emissions in Jilin Province are worked out by specifying the amount of carbon emissions from both planting and animal industries together, as shown in Table 5. The total carbon emissions increased from 11.9451 million tons in 1998 to 12.0803 million tons in 2018, an increase of 135,200 tons. There are not many changes shown in the table, but there is still some fluctuation. A peak appears in 2007, with 15.9076 million tons in total carbon emissions. The main reason for this phenomenon may be the rapid development of animal husbandry and the rapid increase in intestinal and fecal emissions from various animals. Therefore, the overall emissions from animal husbandry are much higher than those from farming, among which animal husbandry accounts for more than 70% of the total agricultural carbon emissions.

**Figure 1:**
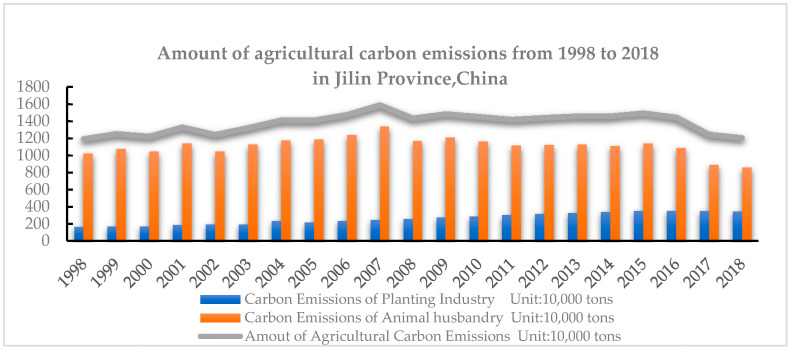
Amount of agricultural carbon emissions from 1998 to 2018 in Jilin Province, China.

To more clearly clarify the changes and trends in the total agricultural carbon emissions in Jilin Province of China from 1998 to 2018, according to the calculation results in Table 3, Table 4 and Table 5, Figure 1 was drawn.

Figure 1 shows changes and trends in agricultural carbon emissions from 1998 to 2018 in Jilin Province. Jilin Province’s total agricultural carbon emission growth can be roughly divided into three stages. The first stage is from 1998 to 2007, which was a period of rapid growth of carbon emissions, with an increase from 11.9451 million tons to 15.9078 million tons, a growth rate of 33.2%, during this period; the highest carbon emissions reached 15,907,800 tons in 2007. It can be seen that at this stage, extensive development was still the main mode in Jilin Province. In the meantime, the central government issued a series of preferential agricultural policies, such as reducing taxes, increasing subsidies, etc., which enhanced the enthusiasm of farmers toward production to a certain extent. It is obvious that the input of agricultural resources, such as fertilizers and pesticides, in the planting industry increased, and the proportion of carbon emissions caused by animal husbandry increased as well, accounting for up to more than 83%. It is also obvious that environmental pollution did not get enough attention, and there was poor awareness of the development of low-carbon agriculture. In the second stage, from 2008 to 2015, agricultural carbon emissions in Jilin Province reached a relatively stable situation, with the overall level increasing slowly, and agricultural carbon emissions increased from 14.362 million tons to 14.9502 million tons, an increase of 0.5882 million tons. The possible reason for this phenomenon was probably rapid development of the planting industry in Jilin Province. The scale of cultivation expanded, but the state and the Jilin provincial government issued a low-carbon development, green development policy at the same time, and people gradually started paying attention to environmental protection. Although the scale of cultivation expanded, the increase in carbon emissions was limited. Carbon emissions from livestock farming also declined, accounting for more than 76% of the total carbon emissions. In the third stage, from 2016 to 2018, there was an overall downward trend on the whole, with the total carbon emissions declining from 14.4697 million tons to 12.0803 million tons, a decrease of 2.3894 million tons, or 16.5%. It can also be seen that the concept and policy of carbon emission reduction achieved certain results.

The conclusion is consistent with Huang (2019) [9], who found that the total agricultural carbon emissions of 31 provinces and cities in China from 1997 to 2016 showed an upward trend on the whole.

**Figure 2:**
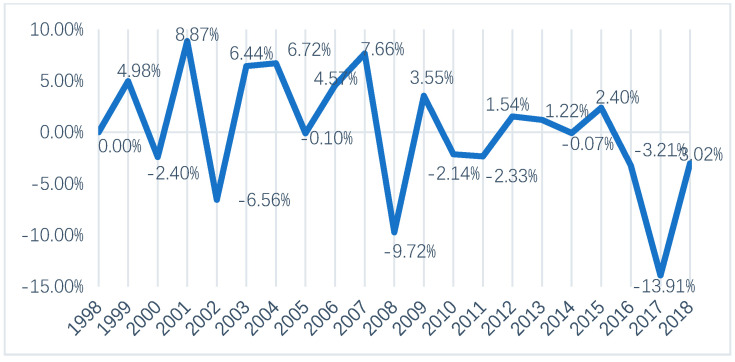
The sequential growth rate of total agricultural carbon emissions in Jilin Province, China.

Figure 2 shows the sequential growth rate of total carbon emissions. From the perspective of the sequential growth rate, the growth rate of agricultural carbon emissions in Jilin Province changed by a large margin, and the inter-annual change in the sequential growth rate showed fluctuations. The sequential growth rate reached a maximum of 8.87% in 2001, and in 2017 the sequential growth rate was the lowest in history, at −13.91%. In fact, this is not the first time that a negative value occurred; as early as in 2000, 2002, 2005, and 2008, the sequential growth rate of agricultural carbon emissions was negative, too, and various natural disasters, such as floods, droughts, wind, and hail, led to a significant reduction in the input of agricultural chemicals, thus causing changes in the total agricultural carbon emissions. The sequential growth rate from 2009 to 2018 showed a fluctuating trend. In particular, from 2016 to 2018, the sequential growth rate was negative each year, at −3.21%, −13.91%, and −3.02%, respectively. This implies that such measures worked well in Jilin Province with respect to the development of the agricultural economy and other aspects, the promotion of a policy to return farmlands to forests and grass, and the influence of green development and sustainable development as well. However, given that carbon emissions in Jilin Province are still at a high level overall, the government and the public should continue to focus on carbon emission reduction, which is a necessary condition for the realization of low-carbon agriculture and green agriculture.

#### 3.1.3. Analysis of Dynamic Changes in Agricultural Carbon Emission Intensity and Density

**Figure 3:**
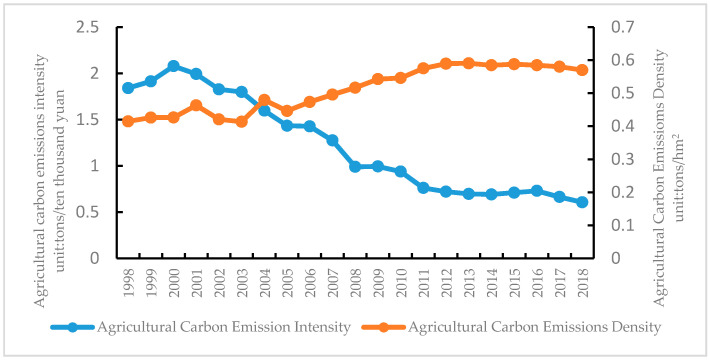
Intensity and density of agricultural carbon emissions in Jilin Province, China.

To further study the dynamic changes in agricultural carbon emissions’ intensity and density in Jilin Province, an analysis was performed according to Equations (4) and (5), as shown in Figure 3.

Figure 3 (left) shows changes in the agricultural carbon emission intensity, and Figure 3 (right) shows changes in the agricultural carbon emission density. Comparing the carbon emission intensities due to the agricultural modernization of Jilin Province from 1998 to 2018, we observed a volatility trend of first increasingand then decreasing. The carbon emissions declined from 1.84 tons/10,000 yuan in 1998 to 0.61 tons/10,000 yuan in 2018. In 2000, the maximum agricultural carbon emission intensity was 2.08 tons/10,000 yuan. The carbon intensity declined from 2000 to 2015, possibly due to the advances in agricultural technology. In particular, since 2007, the annual carbon emission intensity in Jilin Province has been less than the 20-year average, which also indicates that the low-carbon emission effects in Jilin Province related to agriculture in recent years have been more significant. From the perspective of the agricultural carbon emission density in Jilin Province, we observed an evolutionary process of increasing, decreasing, and then increasing, a trend of fluctuating growth, from 0.41 tons/hectare in 1998 to 0.57 tons/hectare in 2018. In 2013, the agricultural carbon emission density reached the maximum value of 0.59 tons/hectare in the entire research process. The carbon emission density of the planting industry changes with a changing in the crop-planting area, rice-planting area, and total carbon emissions.

The conclusion is consistent with Huang (2019) [9], who found that the agricultural carbon emission intensity in China showed a downward trend from 1997 to 2016. The above research indicates that the changing trend in the agricultural carbon emission intensity and density in Jilin Province is a typical type of low-intensity, high-density category, which confirms that there is an improvement in the modernization level of agricultural in Jilin Province to a certain extent, promoting the increase in agricultural carbon emissions, and the agricultural carbon emissions may continue to increase in the future with the vigorous promotion of projects of ten billion pounds of grain production in Jilin Province. Therefore, the development of both sustainable agriculture and low-carbon agriculture is the key to the future development of the agricultural industry in Jilin Province.

### 3.2. Analysis of Influential Factors in Agricultural Carbon Emissions in Jilin Province

According to the LMDI model, the influential factors in agricultural carbon emissions can be analyzed by using Equations (7)–(12). An in-depth analysis of the impacts of various factors contributing to changes in agricultural carbon emissions and the decomposition results of agricultural carbon emissions in Jilin Province are shown in Table 6. Based on the results of LMDI analysis, the changes in carbon emissions caused by the changes in various influential factors are shown as positive values, indicating that the influential factors have a positive effect on the changes in carbon emissions; on the contrary, if the change in carbon emissions are shown as negative value, indicating that the influencial factors have a negative impact on the change in carbon emissions. Based on what is mentioned above, the results fall into two categories: one is the inhibitory factor of agricultural carbon emissions, and the other is the driving factor of agricultural carbon emissions.

Based on the analysis of the influential factors in agricultural carbon emissions in Jilin Province, and by comparing the results with the conclusion by Tian (2014) [8], we concluded that efficiency factors, structural factors, and labor factors all suppressed China’s agricultural carbon emissions to a certain degree, while the rapid improvement of the economic level led to a continuous increase in agricultural carbon emissions.

#### 3.2.1. Agricultural Productivity Factors

Agricultural productivity is the most significant factor that curbs agricultural carbon emissions. The effects of production efficiency factors on agricultural carbon emissions in Jilin Province are wholly negative, except for the positive values shown for a few years. This also achieved a cumulative reduction in agricultural carbon emissions in Jilin Province over the past 20 years to 13.4835 million tons, which also implies that assumption that the other three influential factors remain unchanged. There is still some improvement in the agricultural productivity in Jilin Province, a development caused by the advancement in and innovation of scientific and technological knowledge. In addition, the further upgrade in agricultural modernization has facilitated a decrease in carbon emissions in Jilin Province to an average of 0.6742 million tons per year over the past 20 years. This inhibitory effect was the largest in 2008, with a cumulative inhibition of 3.0679 million tons. However, since 2011, the overall degree of inhibition has decreased slightly compared to previous years. This indicates that improving agricultural production conditions and promoting the advancement in agricultural science and technology are necessary to reduce agricultural carbon emissions in Jilin Province, which is one of the key tasks to achieve sustainable development of agriculture in Jilin Province in the future.

#### 3.2.2. Agricultural Industrial Structure Factors

Table 6 indicates that compared to the factors in agricultural productivity and agricultural labor force, the overall degree of inhibition is relatively slight in agricultural industrial structure factors, showing dynamic changes as a whole, with a slight fluctuation of the curve. A total of 778,900 tons of carbon emission reduction has been achieved by agricultural structural factors over the past 20 years, of which 495,900 tons of carbon emissions were inhibited in 2002, reaching a maximum value in history during the research period. Given that other influential factors remain unchanged, an average of 38,900 tons of carbon emission reduction has been achieved each year. Carbon emissions show positive values in some years and negative values in other years. These results also indicate that the agricultural industrial structure of Jilin Province has been in the process of adjustment and transformation to a certain extent, which has led to unstable results of the proportion of the agricultural industrial structure factors in agriculture carbon emissions. The ratio of the total output value of agriculture, forestry, animal husbandry, and fishery in Jilin Province increased from 388.4:10.45:266.84:9.61 in 1999 to 992.96:73.28:1001.64:39.02 in 2018. Although the output value of forestry and fisheries has improved to a certain extent, the agricultural industrial structure of Jilin Province is still dominated by cultivation and animal husbandry, accounting for 45% and 46%, respectively, of the total output value of agriculture, forestry, animal husbandry, and fishery. Therefore, with the promotion of supply structural reforms and the development of sustainable agriculture, there is a need to give full play to the role of cultivation and animal husbandry; further optimize the industrial structure; promote the comprehensive and coordinated development of agriculture, forestry, animal husbandry, and fishery; and explore the new low-carbon agricultural development model of high output and low emissions.

#### 3.2.3. Agricultural Labor Force Factors

The agricultural labor force factor means the degree of change in agricultural carbon emissions caused by an increase or a decrease in the number of agricultural workers. As one of the main kinetic factors in social productivity, agricultural labor force factors have a significant influence on agricultural input, production models, and management and decision making for the agricultural labor force to a large extent. It can be seen from the figure and table that the agricultural labor force factor is the indirect inhibitory factor in the agricultural carbon emissions, and the effect of the inhibitory factor on the growth of carbon emissions lies between the agricultural carbon intensity factors and the agricultural industrial structure factors. In total, 1.5755 million tons of carbon emissions were inhibited from 1999 to 2018, given that other influential factors remained unchanged, that is, an average of 78,800 tons of carbon emissions were inhibited each year. With the increase in urbanization in Jilin Province, the number of agricultural laborers moving to the secondary and tertiary industry has increased as well, and the changing number of employees has reduced the size of the labor force, which can inhibit carbon emissions from the source to a certain extent. In addition, by improving the quality of the agricultural labor force and allowing agricultural practitioners to master knowledge of scientific agricultural management and agriculture-related skills, which improves the agricultural production efficiency, and decreases carbon emissions to a certain extent as well.

#### 3.2.4. Economic Factor

Table 6 indicates that the economic factor is the main driving factor that increases agricultural carbon emissions in Jilin Province. It shows dynamic changes on the whole, with relatively greater fluctuations. It played a role in reducing carbon emissions during 1999–2000, 2015–2016, and 2016–2017 and showed a positive effect in the remaining time periods. It contributed a cumulative total of 15.9659 million tons of carbon emissions in Jilin Province from 1999 to 2018, contributing an average of 0.7983 million tons per year over the past 20 years, while other factors remained unchanged. The contribution reached the maximum value of 2.9528 million tons of carbon emissions from 2003 to 2004. This was mainly due to the implementation of a preferential agricultural policy and an increase in the agricultural income, which increased the enthusiasm of farmers toward production, which simultaneously led to a further expansion of the agricultural production scale and increased the inputs of agricultural resources, such as pesticides, fertilizers, etc. Though it increased the economic efficiency, it also caused high agricultural carbon emissions at the same time. From the current point of view, the agricultural economic development mode currently applied in Jilin Province is still a high-carbon-based model, so it will still lead to an increase in carbon emissions in the short term. Therefore, how to reduce agricultural carbon emissions and realize the development of low-carbon agriculture and sustainable agriculture truly, while maintaining economic growth in Jilin Province, China, is still an issue to be solved.

### 3.3. Policy Implication

Based on the above results, to better realize the low-carbon development of agriculture in Jilin Province, this study put forward the following countermeasures and suggestions.

First, it is necessary to improve the policy of low-carbon agriculture. On the one hand, economic policies such as the low-carbon agricultural subsidy mechanism, tax relief, carbon trust fund, and financial credit support should be established to reward farmers, enterprises, and collectives who have made outstanding contributions to carbon emission reduction. On the other hand, the government should also strengthen the supervision of agricultural carbon emissions and establish and improve relevant policies and regulations, such as limiting the use of agricultural chemical fertilizers and other high-carbon agricultural means of production, establishing a carbon tax system, and so on.

Second, the industrial structure and the mode of agricultural production should be adjusted. At present, the mode of agricultural production is still in an outdated and extensive state, economic development is the driving factor for an increase in carbon emissions, and there are still many problems such as low level of technology. More measures should be taken on the basis of reasonably reducing agricultural material input and using organic fertilizers instead of chemical fertilizers, drawing lessons from the more successful low-carbon production models, such as circular agriculture, water-saving agriculture, etc., to improve the level of agricultural modernization. At the same time, optimizing the industrial structure is the main developmental strategy of Jilin Province, which vigorously promotes the developmental structure of the combination of planting and breeding. In this way, it can not only solve the problem of carbon emissions caused by livestock excrement but also reduce the input of agricultural materials. Agricultural waste resources can also be deployed. Therefore, the development of sustainable agricultural can be realized only when the extensive mode of high pollution and high emission is changed into the intensive mode of low-energy consumption and high value addition.

Third, the level of agricultural science and technology should be enhanced and new technologies should be actively introduced. New methods such as three-dimensional culture to replace traditional planting and high-light-efficiency cultivation techniques to improve rice production can be implemented so as to really keep the production benefit as the focus and consider the economic benefit as the driving force for the low-carbon supply of agriculture. There is also manure methane extraction technology to be considered, which could maximize the use of methane as renewable energy for power generation. On the one hand, it can promote the development of low-carbon agriculture; on the other hand, it can promote the development of a low-carbon economy.

Fourth, publicity and education should be further reinforced to cultivate innovative talents in agricultural science and technology. This aims to raise people’s awareness of low-carbon agriculture by organizing study tours and other activities. Through training and education of farmers’ production technology and the use of production equipment, farmers are encouraged to master more agricultural production technology, which can not only improve labor production efficiency but also reduce agricultural carbon emissions.

## 4. Conclusions

The following are the conclusions of the paper:

(1) From 1998 to 2018, agricultural carbon emissions in Jilin Province of China showed an upward trend as a whole, but the growth rate slowed down. From the perspective of a sequential growth rate, there is a significant range of variation, and the inter-annual variation of the sequential growth rate shows fluctuation. In recent years, the sequential growth rate has been negative. Agricultural carbon emissions in Jilin Province belong to the low-intensity, high-density category. From the perspective of animal husbandry, it is still the main cause of agricultural carbon emissions. CH_4_ and N_2_O are the two main greenhouse gases in Jilin Province at present. The proportion of CH_4_ is shrinking day by day. The proportion of N_2_O is relatively stable. In terms of the planting industry, the input of agricultural resources is still the main source of carbon emissions. The increase in carbon emissions from rice planting is mainly affected by the expansion of the planting scale and planting structure, which leads to an increase in carbon emissions from rice planting.

(2) Based on the results of LMDI decomposition, production efficiency factors, industrial structure factors, and labor factors have a strong inhibitory effect on agricultural carbon emissions in Jilin Province. Of these, production efficiency is the most important factor that restrains agricultural carbon emissions. However, economic factors have an obvious promotional effect on carbon emissions. At present, the mode of agricultural economic development in Jilin Province is still high carbon, and the transformation of the agricultural production mode is the key to achieving agricultural carbon emission reduction in Jilin Province.

Compared to existing research, this paper discusses the selection of carbon sources more comprehensively and in detail. The planting industry is not the only thing to be considered but the combination of planting and animal husbandry, covering a relatively wider and specific scope. However, there are certain limitations to this paper. It estimates the differences in agricultural carbon emissions only with regard to the aspects of total carbon emissions, structure, carbon intensity and density, and influential factors. This paper does not carefully consider the factor of straw burning, and there may be other potential factors affecting agricultural carbon emissions. The carbon emission efficiency is also a very significant reference indicator, and all these need to be considered in further follow-up studies.

## Figures and Tables

**Table 1:** Carbon emission coefficients of major agricultural sources.

Carbon Sources	Carbon Emission Coefficient	References
Fertilizer	0.8956 kg (C)/kg	[42,43,44]
Agricultural diesel fuel	0.5927 kg (C)/kg	[45]
Pesticide	4.9341 kg (C)/kg	[42,46,47]
Agricultural plastic film	5.18 kg (C)/kg	[9,48,49,50]
Land plowing	3.126 kg (C)/hm^2^	[51]
Irrigation	266.48 kg (C)/hm^2^	[52]
Paddy rice planting	66.2 kg CH_4_/hm^2^	[41]

**Table 2:** Carbon emission coefficients of major livestock in Jilin Province (unit: kg/head/year).

Sources	Emission Factors in Intestinal Fermentation	Emission Factors in Manure Treatment	
	CH_4_	CH_4_	N_2_O
Cattle	47.80	1	1.39
Horse	18	1.64	1.39
Donkey	10	0.90	1.39
Mule	10	0.90	1.39
Goat	5	0.17	0.33
Sheep	5	0.15	0.33
Pig	1	3.50	0.53

Source: IPCC (Intergovernmental Panel on Climate Change) (2007).

**Table 3:** Carbon emission of the planting industry in Jilin Province from 1998 to 2018 (unit: 10,000 tons).

Year	Fertilizer (Convert into Purification)	Agricultural Diesel Fuel	Pesticide	Agricultural Plastic Film	Land Plowing	Irrigation	Paddy Rice Planting	Total
1998	100.7550	14.6397	8.2360	18.5102	0.1270	0.0333	26.0987	168.3999
1999	104.0687	15.6473	8.7620	18.0559	0.1271	0.0345	26.4513	173.1467
2000	100.3968	16.6549	9.7098	18.8500	0.1271	0.0350	27.5146	173.2882
2001	102.1880	20.0333	11.8907	26.6563	0.1265	0.0369	26.4626	187.3942
2002	104.7852	19.2035	11.7190	23.4411	0.1465	0.0399	37.8744	197.2096
2003	109.5319	20.6852	11.7190	22.1668	0.1475	0.0412	30.7613	195.0528
2004	142.4900	23.9451	12.6752	21.6773	0.1533	0.0425	34.1217	235.1050
2005	123.6824	24.0044	14.2566	21.6255	0.1548	0.0430	37.1864	220.9530
2006	131.3845	25.9603	16.9985	23.6312	0.1558	0.0436	37.7550	235.9289
2007	138.2806	29.9314	18.6104	24.4755	0.1574	0.0437	38.1815	249.6806
2008	146.6993	31.5909	19.9959	25.9554	0.1587	0.0447	37.8403	262.2854
2009	156.0135	34.1988	20.9078	26.9256	0.1591	0.0449	37.9541	276.2038
2010	163.7157	36.1547	21.1101	27.2219	0.1644	0.0460	38.6762	287.0890
2011	174.8211	37.8735	22.4970	29.5617	0.1656	0.0488	39.6712	304.6390
2012	185.1205	39.5924	25.2818	29.3706	0.1698	0.0493	40.4616	320.0460
2013	194.1661	40.7185	25.1693	30.2952	0.1761	0.0494	42.0423	332.6169
2014	203.0325	38.7033	29.3663	29.9704	0.1841	0.0434	43.0430	344.3431
2015	207.0627	39.5331	30.7320	30.6470	0.1875	0.0477	44.2826	352.4926
2016	209.2122	39.7109	28.8758	30.8547	0.1895	0.0488	45.4994	354.3913
2017	206.8836	40.0665	27.7760	31.4695	0.1903	0.0526	46.6707	353.1092
2018	204.4655	39.8887	25.1595	29.1199	0.1901	0.0512	47.7453	346.6202

Data Source: Calculated by the authors.

**Table 4:** Carbon emission from animal husbandry in Jilin Province from 1998 to 2018 (unit: 10,000 tons).

Year	Methane (CH_4_) Carbon Emission		Nitrous Oxide (N_2_O) Emission	Total
	Intestinal Fermentation in Animals	Manure Treatment Emission from Animals	Manure Treatment Emission from Animals	
1998	22.5889	3.5792	1.2480	1026.1109
1999	24.0032	3.6887	1.3038	1080.8206
2000	24.7119	2.8975	1.2092	1050.5638
2001	25.8800	3.7562	1.3559	1144.9773
2002	25.8480	2.3162	1.1533	1047.7888
2003	27.8931	2.5308	1.2402	1130.1742
2004	29.0769	2.6996	1.2914	1179.2391
2005	29.1241	2.8648	1.3164	1192.0038
2006	30.3673	2.9973	1.3676	1241.6583
2007	30.2897	4.5101	1.5808	1341.0771
2008	26.2059	4.0481	1.4012	1173.9110
2009	27.0105	4.1776	1.4475	1211.0412
2010	25.9485	4.0786	1.4018	1168.4021
2011	24.3984	4.0557	1.3608	1116.8676
2012	24.6174	4.0891	1.3616	1123.4207
2013	24.8311	4.0869	1.3606	1128.3932
2014	24.4902	4.0711	1.3478	1115.6921
2015	25.4882	3.9873	1.3612	1142.5308
2016	24.2416	3.8738	1.3076	1092.5797
2017	19.1120	3.5933	1.0903	892.5644
2018	18.4791	3.4392	1.0518	861.4138

Data Source: Calculated by the authors.

**Table 5:** Amount of agricultural carbon emissions in Jilin Province from 1998 to 2018 (unit: 10,000 tons).

Year	Planting Industry	Animal Husbandry	Total
1998	168.3999	1026.1109	1194.5108
1999	173.1467	1080.8206	1253.9672
2000	173.2882	1050.5638	1223.8519
2001	187.3942	1144.9773	1332.3715
2002	197.2096	1047.7888	1244.9984
2003	195.0528	1130.1742	1325.2269
2004	235.1050	1179.2391	1414.3441
2005	220.9530	1192.0038	1412.9569
2006	235.9289	1241.6583	1477.5872
2007	249.6806	1341.0771	1590.7576
2008	262.2854	1173.9110	1436.1963
2009	276.2038	1211.0412	1487.2449
2010	287.0890	1168.4021	1455.4911
2011	304.6390	1116.8676	1421.5066
2012	320.0460	1123.4207	1443.4668
2013	332.6169	1128.3932	1461.0101
2014	344.3431	1115.6921	1460.0352
2015	352.4926	1142.5308	1495.0234
2016	354.3913	1092.5797	1446.9710
2017	353.1092	892.5645	1245.6737
2018	346.6202	861.4139	1208.0341

Data Source: Calculated by the authors.

**Table 6:** Influential factors in agricultural carbon emissions in Jilin Province from 1998 to 2018.

Year	Productivity Factors ΔEI	Industrial Structure ΔSI	Economic Factor ΔCI	Labor Force Factors ΔAI	Total
1998–1999	48.3896	−4.3646	2.7186	13.5282	60.2718
1999–2000	99.7914	−4.6601	−196.8514	71.2459	−30.4741
2000–2001	−45.2038	−0.5016	149.9128	7.0462	111.2537
2001–2002	−115.9615	−49.5915	71.5304	−0.1649	−94.1875
2002–2003	−13.8024	0.8269	86.9847	13.7638	87.7730
2003–2004	−159.0555	13.5501	295.2792	−62.2688	87.5050
2004–2005	−144.0494	−3.8297	138.5485	5.7550	−3.5755
2005–2006	−14.4123	−10.4770	89.9131	−0.2906	64.7331
2006–2007	−171.5450	8.4093	275.6514	0.4042	112.9199
2007–2008	−306.7920	5.2773	152.1877	−3.9016	−153.2285
2008–2009	−9.5718	−24.8521	73.2682	12.2353	51.0797
2009–2010	−65.6909	−8.4765	46.8752	−3.9052	−31.1973
2010–2011	−265.4066	5.0139	213.9249	14.4075	−32.0603
2011–2012	−81.0125	−4.5507	146.5715	−39.1709	21.8375
2012–2013	−53.5860	4.1737	78.9972	−13.3337	16.2511
2013–2014	−14.3466	−3.8750	54.2043	−43.7565	−7.7739
2014–2015	22.6415	−1.2047	38.6042	−21.8024	38.2385
2015–2016	58.4733	−4.2848	−58.8264	−43.3717	−48.0096
2016–2017	−25.4118	−0.1788	−138.7968	−34.2008	−198.5882
2017–2018	−91.7980	5.7097	75.8975	−29.7416	−39.9323

Data Source: Calculated by the authors. EI represents agricultural production efficiency indicators; SI represents agricultural structural factors; CI rep-resents agricultural economic development-level factors; and AI represents agricultural labor factors.

## Data Availability

Publicly available datasets were analyzed in this study. This data can be found here: China Statistical Yearbook (http://www.stats.gov.cn/english/Statisticaldata/AnnualData/), China Rural Statistical Yearbook (https://data.cnki.net/trade/yearbook/single/n2019120190?z=z009), and Jilin Statistical Yearbook (http://tjj.jl.gov.cn/tjsj/tjnj/2019/ml/indexe.htm).

## References

[1] Bai Y., Deng X., Gibson J., Zhao Z., Xu H. (2019). How does urbanization affect residential CO2 emissions? An analysis on urban agglomerations of China. J. Clean. Prod..

[2] Qiu H., Hu G., Yang Y., Zhang J., Zhang T. (2020). Modeling the Risk of Extreme Value Dependence in Chinese Regional Carbon Emission Markets. Sustainability.

[3] Li L., Lei Y., He C., Wu S., Chen J. (2016). Prediction on the Peak of the CO_2_ Emissions in China Using the STIRPAT Model. Adv. Meteorol..

[4] IPCC (1996). Climate Change 1995—Impacts, Adaptations and Mitigation of Climate Change: Scientific-Technical Analyses.

[5] Wang H., Yang Y., Zhang X., Tian G. (2015). Carbon Footprint Analysis for Mechanization of Maize Production Based on Life Cycle Assessment: A Case Study in Jilin Province, China. Sustainability.

[6] He Y., Chen R., Wu H., Xu J., Song Y. (2018). Spatial dynamics of agricultural carbon emissions in China and the related driving factors. Chin. J. Eco-Agric..

[7] Dalgaard T., Olesen J.E., Petersen S.O., Petersen B.M., Jorgensen U., Kristensen T., Hutchings N.J., Gyldenkaerne S., Hermansen J.E. (2011). Developments in greenhouse gas emissions and net energy use in Danish agriculture—How to achieve substantial CO(2) reductions?. Environ. Pollut..

[8] Tian Y., Zhang J.-B., He Y.-Y. (2014). Research on Spatial-Temporal Characteristics and Driving Factor of Agricultural Carbon Emissions in China. J. Integr. Agric..

[9] Huang X., Xu X., Wang Q., Zhang L., Gao X., Chen L. (2019). Assessment of Agricultural Carbon Emissions and Their Spatiotemporal Changes in China, 1997–2016. Int. J. Environ. Res. Public Health.

[10] Garnier J., Le Noe J., Marescaux A., Sanz-Cobena A., Lassaletta L., Silvestre M., Thieu V., Billen G. (2019). Long-term changes in greenhouse gas emissions from French agriculture and livestock (1852–2014): From traditional agriculture to conventional intensive systems. Sci. Total Environ..

[11] Luo X., Ao X., Zhang Z., Wan Q., Liu X. (2020). Spatiotemporal variations of cultivated land use efficiency in the Yangtze River Economic Belt based on carbon emission constraints. J. Geogr. Sci..

[12] Bennetzen E.H., Smith P., Porter J.R. (2016). Decoupling of greenhouse gas emissions from global agricultural production: 1970–2050. Glob. Chang. Biol..

[13] Peter C., Helming K., Nendel C. (2017). Do greenhouse gas emission calculations from energy crop cultivation reflect actual agricultural management practices?—A review of carbon footprint calculators. Renew. Sustain. Energy Rev..

[14] Linderholm K., Katterer T., Mattsson J.E. (2020). Valuing carbon capture in agricultural production: Examples from Sweden. SN Appl. Sci..

[15] Lu X., Kuang B., Li J., Han J., Zhang Z. (2018). Dynamic Evolution of Regional Discrepancies in Carbon Emissions from Agricultural Land Utilization: Evidence from Chinese Provincial Data. Sustainability.

[16] Czubaszek R., Wysocka-Czubaszek A. (2018). Emissions of carbon dioxide and methane from fields fertilized with digestate from an agricultural biogas plant. Int. Agrophysics.

[17] Wang G., Liao M., Jiang J. (2020). Research on Agricultural Carbon Emissions and Regional Carbon Emissions Reduction Strategies in China. Sustainability.

[18] Ridzuan N.H.A.M., Marwan N.F., Khalid N., Ali M.H., Tseng M.L. (2020). Effects of agriculture, renewable energy, and economic growth on carbon dioxide emissions: Evidence of the environmental Kuznets curve. Resour. Conserv. Recycl..

[19] Fernández González P., Landajo M., Presno M.J. (2014). The driving forces behind changes in CO_2_ emission levels in EU-27. Differences between member states. Environ. Sci. Policy.

[20] Alamdarlo H.N. (2016). Water consumption, agriculture value added and carbon dioxide emission in Iran, environmental Kuznets curve hypothesis. Int. J. Environ. Sci. Technol..

[21] Javid M., Sharif F. (2016). Environmental Kuznets curve and financial development in Pakistan. Renew. Sustain. Energy Rev..

[22] Zhang P.Y., He J.J., Hong X., Zhang W., Qin C.Z., Pang B., Li Y.Y., Liu Y. (2017). Regional-Level Carbon Emissions Modelling and Scenario Analysis: A STIRPAT Case Study in Henan Province, China. Sustainability.

[23] Tongwane M.I., Moeletsi M.E., Tsubo M. (2020). Trends of carbon emissions from applications of nitrogen fertiliser and crop residues to agricultural soils in South Africa. J. Environ. Manag..

[24] Coderoni S., Esposti R. (2011). Is there a Long-Term Relationship between Agricultural GHG Emissions and Productivity Growth? The Case of Italian Agriculture. Work. Pap..

[25] Yadav D., Wang J. (2017). Modelling carbon dioxide emissions from agricultural soils in Canada. Environ. Pollut..

[26] Baumann M., Gasparri I., Piquer--Rodríguez M., Gavier Pizarro G., Griffiths P., Hostert P., Kuemmerle T. (2017). Carbon emissions from agricultural expansion and intensification in the Chaco. Glob. Chang. Biol..

[27] Liu X., Zhang S., Bae J. (2017). The impact of renewable energy and agriculture on carbon dioxide emissions: Investigating the environmental Kuznets curve in four selected ASEAN countries. J. Clean. Prod..

[28] Zafeiriou E., Mallidis I., Galanopoulos K., Arabatzis G. (2018). Greenhouse Gas Emissions and Economic Performance in EU Agriculture: An Empirical Study in a Non-Linear Framework. Sustainability.

[29] Gokmenoglu K.K., Nigar T. (2018). Testing the agriculture-induced EKC hypothesis: The case of Pakistan. Environ. Sci. Pollut. Res..

[30] Zhang Q., Xiao J., Xue J., Zhang L. (2020). Quantifying the Effects of Biochar Application on Greenhouse Gas Emissions from Agricultural Soils: A Global Meta-Analysis. Sustainability.

[31] Coderoni S., Esposti R. (2018). CAP payments and agricultural GHG emissions in Italy. A farm-level assessment. Sci. Total Environ..

[32] Benbi D.K. (2018). Carbon footprint and agricultural sustainability nexus in an intensively cultivated region of Indo-Gangetic Plains. Sci. Total Environ..

[33] Diacono M., Persiani A., Testani E., Montemurro F., Ciaccia C. (2019). Recycling Agricultural Wastes and By-products in Organic Farming: Biofertilizer Production, Yield Performance and Carbon Footprint Analysis. Sustainability.

[34] Li J., Luo Z., Wang Y., Li H., Xing H., Wang L., Wang E., Xu H., Gao C., Ren T. (2019). Optimizing Nitrogen and Residue Management to Reduce GHG Emissions while Maintaining Crop Yield: A Case Study in a Mono-Cropping System of Northeast China. Sustainability.

[35] Qin J., Tao H., Cheng C., Brindha K., Zhan M., Ding J., Mu G. (2020). Analysis of Factors Influencing Carbon Emissions in the Energy Base, Xinjiang Autonomous Region, China. Sustainability.

[36] Peng H., Wang Y., Hu Y., Shen H. (2020). Agglomeration Production, Industry Association and Carbon Emission Performance: Based on Spatial Analysis. Sustainability.

[37] Xiong C., Chen S., Xu L. (2020). Driving factors analysis of agricultural carbon emissions based on extended STIRPAT model of Jiangsu Province, China. Growth Chang..

[38] Abbas F., Hammad H.M., Ishaq W., Farooque A.A., Bakhat H.F., Zia Z., Fahad S., Farhad W., Cerda A. (2020). A review of soil carbon dynamics resulting from agricultural practices. J. Environ. Manag..

[39] Dumortier J., Dokoohaki H., Elobeid A., Hayes D.J., Laird D., Miguez F.E. (2020). Global land-use and carbon emission implications from biochar application to cropland in the United States. J. Clean. Prod..

[40] Ma Q., Li J., Aamer M., Huang G. (2020). Effect of Chinese Milk Vetch (*Astragalus sinicus* L.) and Rice Straw Incorporated in Paddy Soil on Greenhouse Gas Emission and Soil Properties. Agronomy.

[41] Tian Y., Zhang J.B. (2013). Study on the differentiation of net carbon effect of Agricultural production in China. J. Nat. Resour..

[42] Shu-jie Y., Yu-bo L., Shou-gang Y. (2018). An Empirical Analysis of the Decoupling Relationship between Agricultural Carbon Emission and Economic Growth in Jilin Province. IOP Conf. Ser. Mater. Sci. Eng..

[43] Sun Q. (2019). Ecological agriculture development and spatial and temporal characteristics of carbon emissions of land use. Appl. Ecol. Environ. Res..

[44] Liu X.Y., Yu Y.L., Luan S.J. (2019). Empirical Study on the Decomposition of Carbon Emission Factors in Agricultural Energy Consumption. IOP Conf. Ser. Earth Environ. Sci..

[45] (2019). Intergovernmental Panel on Climate Change. Special Report on Climate Change and Land. Proceedings of the Intergovernmental Panel on Climate Change (IPCC).

[46] Chen Y., Li M., Su K., Li X. (2019). Spatial-Temporal Characteristics of the Driving Factors of Agricultural Carbon Emissions: Empirical Evidence from Fujian, China. Energies.

[47] Zhang L., Pang J., Chen X., Lu Z. (2019). Carbon emissions, energy consumption and economic growth: Evidence from the agricultural sector of China’s main grain-producing areas. Sci. Total Environ..

[48] Chen X., Shuai C., Wu Y., Zhang Y. (2020). Analysis on the carbon emission peaks of China’s industrial, building, transport, and agricultural sectors. Sci. Total Environ..

[49] Akiyama H., Yan X., Yagi K. (2009). Evaluation of effectiveness of enhanced-efficiency fertilizers as mitigation options for N_2_O and NO emissions from agricultural soils: Meta-analysis. Glob. Chang. Biol..

[50] Balsalobre-Lorente D., Driha O.M., Bekun F.V., Osundina O.A. (2019). Do agricultural activities induce carbon emissions? The BRICS experience. Environ. Sci. Pollut. Res. Int..

[51] Wu F.L., Li L., Zhang H.L., Chen F. (2007). Effect of conservation tillage on net carbon release from farmland ecosystem. Chin. J. Ecol..

[52] West T.O., Marland G. (2002). A synthesis of carbon sequestration, carbon emissions, and net carbon flux in agriculture: Comparing village practices in U.S.. Agric. Ecosyst. Environ..

[53] IPCC (2014). Climate Change 2014: Synthesis Report. Contribution of Working Groups I, II and III to the Fifth Assessment Report of the Intergovernmental Panel on Climate Change.

[54] Mahony T.O. (2013). Decomposition of Ireland’s carbon emissions from 1990 to 2010: An extended Kaya identity. Energy Policy.

